# First person – Yoshiaki Kitsu

**DOI:** 10.1242/bio.060578

**Published:** 2024-06-17

## Abstract

First Person is a series of interviews with the first authors of a selection of papers published in Biology Open, helping researchers promote themselves alongside their papers. Yoshiaki Kitsu is first author on ‘ microRNA-guided immunity against respiratory virus infection in mammalian lung cells’, published in BiO. Yoshiaki is a Prospective Graduate Student in the lab of Tomoko Takahashi at Saitama University, investigating antiviral defense systems regulated by non-coding RNAs in mammalian cells.


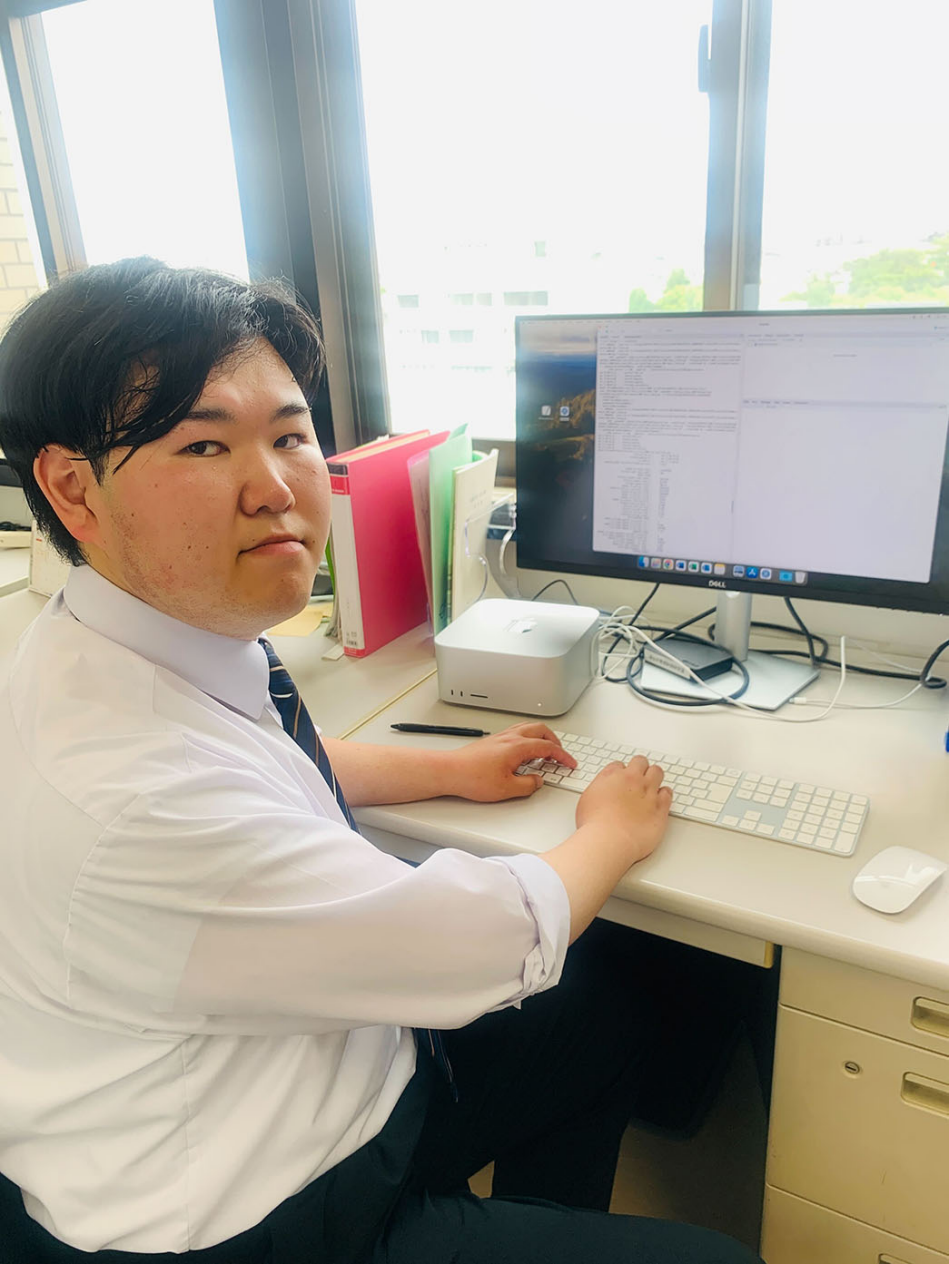


**Yoshiaki Kitsu**

**Describe your scientific journey and your current research focus**

I have a deep interest in the field of non-coding RNAs, particularly their role in the regulation of gene expression during viral infection. During my senior year at Saitama University, I learned bioinformatics and investigated the function of the small non-coding RNAs, microRNAs (miRNAs). This research was incredibly enriching, and I had the opportunity to learn various techniques from my colleague, Ms Shibamoto, who is the co-first author of this paper, and my supervisor, Dr Takahashi.

One of the most intriguing aspects of non-coding RNAs was understanding how, although genes are typically transcribed from DNA to RNA and then translated into proteins, some transcripts exert their functions directly in the RNA state. This discovery sparked my fascination with RNA and motivated me to delve deeper into this area of study. I continuously strive to expand my knowledge and contribute to this exciting field.

**Who or what inspired you to become a scientist?**

I discovered my fascination with biology in a school class when I was 17 years old and decided to pursue research activities in the future. At the time, my greatest interest was genes. I was captivated by the fact that the various elements that make up our bodies are intricately controlled by genes and exist as a single form of life. I believe that there are many mysteries that cannot be solved by one aspect alone. The study of biology, physics, and chemistry, which attempts to unravel these mysteries from a variety of perspectives, is profoundly meaningful to me. Now, I feel honored to have completed our study and to have it published in Biology Open.

I was captivated by the fact that the various elements that make up our bodies are intricately controlled by genes and exist as a single form of life.

**How would you explain the main finding of your paper?**

The pandemic of the new coronavirus has raised global concern about antiviral immunity. Immunity can be divided into cellular and protein-based immunity in mammals, but we have been studying RNA-based antiviral immunity. In this paper, we discuss how its function differs between humans and mice. Our analysis revealed that even for the same viral RNA, the function of antiviral miRNAs may differ between humans and mice. This result may indicate that some differences in antiviral defense system between species originate from miRNAs. We hope that more research will be conducted on other species.

**What are the potential implications of this finding for your field of research?**

I believe that we will learn more about how immune responses differ between different species to the same virus. In addition, we hope that this will lead to the development of novel types of nucleic acid therapeutics to combat viral infections. My colleagues and I believe that we will be able to expand our research to develop these therapeutics, which could save many lives in the future.

**Figure:**
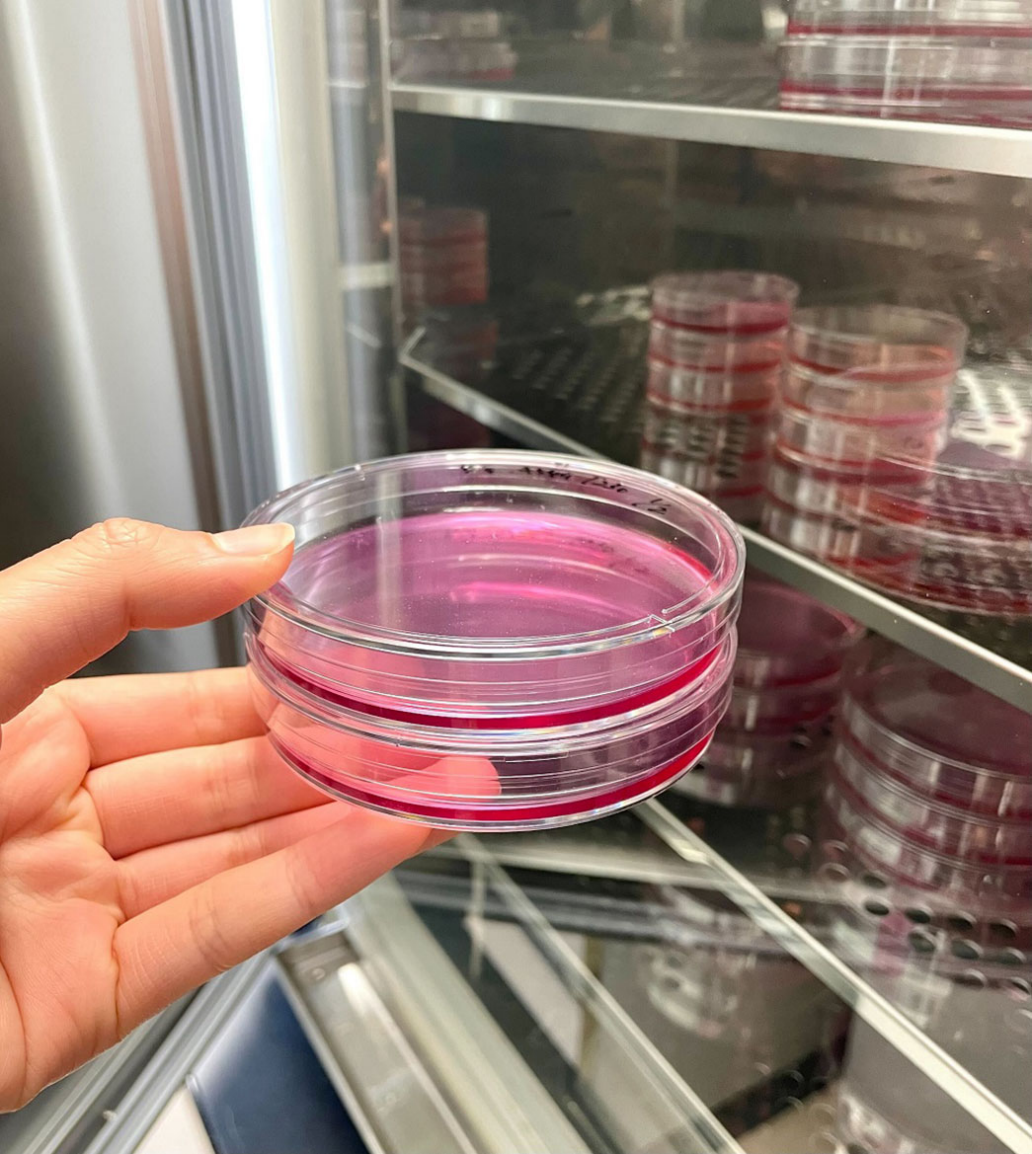
**In our group, we perform both biological experiments and bioinformatics approaches.** I am interested in how non-coding RNAs function as an antiviral defense system in these human cells.

**Which part of this research project was the most rewarding?**

When I started this research, I had no skills in bioinformatics. Therefore, I worked hard to accomplish this research. I was always thinking about where to start and how to proceed, and I repeatedly performed trial-and-error processes.

**What do you enjoy most about being an early-career researcher?**

It takes a lot of courage to venture into uncharted territory, but I enjoy it immensely. I had never tried bioinformatics research before, and this was my first experience with it. I realize that my horizons have been greatly broadened. I believe it is crucial to challenge everything. It is important for young people to boldly try new things without fear of failure.

It takes a lot of courage to venture into uncharted territory, but I enjoy it immensely.

**What piece of advice would you give to the next generation of researchers?**

Don't be afraid to fail and take on challenges!

**What's next for you?**

I would like to resume my research activities and pursue a masters’ or doctoral degree. I aim to continue my research on the antiviral defense system mediated by non-coding RNAs. Regardless of the path I take, I will always maintain an interest in biology and aspire to produce results that will contribute to the future.
